# Numerical Study on the Damage of a Carbon Woven Composite Panel Subjected to Blast Loading

**DOI:** 10.3390/polym15214269

**Published:** 2023-10-30

**Authors:** Alessandro Vescovini, Luca Lomazzi, Marco Giglio, Andrea Manes

**Affiliations:** Politecnico di Milano, Department of Mechanical Engineering, Via La Masa n.1, 20156 Milan, Italy; alessandro.vescovini@polimi.it (A.V.); marco.giglio@polimi.it (M.G.); andrea.manes@polimi.it (A.M.)

**Keywords:** CFRP, blast loading, numerical simulation, reinforced polymers

## Abstract

Blast loading represents a critical dynamic condition for engineering structures. While the response of metal materials to such a condition has been studied in detail, the behavior of composites has not been properly addressed yet. In this context, this work leverages numerical methods to assess the damage that occurs in a carbon-fiber-reinforced polymer plate subjected to close-range blast loading. Numerical analyses were carried out using two methods, i.e., the pure Lagrangian and hybrid coupled Eulerian–Lagrangian approaches. The simulations were validated against observations from a benchmark experimental test taken from the literature. The results showed that (i) the hybrid approach seems to be the most promising solution in terms of efficiency and accuracy; (ii) the Lagrangian approach can accurately reproduce the experimental observations, even though it comes with strong limitations; and (iii) the numerically predicted damage adheres to the experimentally observed damage, although the simulation outcome is influenced by the modeling technique used to describe the behavior of the composite material. We consider the approaches presented in this paper promising for investigation of blast-loaded composite structures, and further improvements can be achieved by (i) refining the description of the material behavior, e.g., by including the strain rate sensitivity; and (ii) better modeling the boundary conditions.

## 1. Introduction

Composite materials are widely used in civil infrastructure, such as bridges and buildings, as well as in military equipment and assets, such as protective panels for armored vehicles, riot shields and military helmets [1,2,3]. When applied in these fields, composites are at risk from blast loading, for instance, from attacks using explosive ordinances. Thus, it is important to investigate the response of composite plate-like structures when subjected to dynamic loading resulting from a blast event.

In recent years, many experimental tests have been carried out to characterize the response of blast-loaded laminates [4,5], including carbon-fiber-reinforced polymer (CFRP) plates [6,7,8]. Such tests are often expensive, time-consuming and potentially dangerous. Moreover, to date, full-field information about the pressure load exerted on blast-loaded structures has not been retrievable through experiments. These limitations, combined with the difficulties determined by the numerous damage mechanisms characterizing composite structures, have led researchers to use high-fidelity numerical simulations to complement experimental observations.

The behavior of composite structures subjected to dynamic loading is often simulated by employing finite-element (FE) analyses [5]. The FE method is a powerful tool to comprehensively account for the non-linear and post-failure behavior of composites, which are crucial to accurately represent the mechanical behavior of these materials [7,9]. However, to the best of our knowledge, only a few numerical models of blast-loaded composite panels presented in the literature have been validated against experimental observations [5]; exceptions are found in the works of Gargano et al. (2019) and Gunaryo et al. (2020) [7,10], where validated FE models of CFRP laminates and woven glass/epoxy composite plates subjected to blast loading were presented, respectively. In addition, most of the contributions involving composite structures are based on uncoupled simulations, where the pressure load from the explosion is described through analytical and/or empirical models. This poses severe challenges in terms of the accurate description of the structural dynamics of blast-loaded composite plates. That is, uncoupled approaches are not able to replicate fluid–structure interaction (FSI) effects [11,12], which have been shown to have a considerable influence on composites [13], and the models employed to predict the pressure load are not valid in some close-range scenarios [14].

This work aims to present evidence that the coupled Eulerian–Lagrangian (CEL) approach can be used to describe blast-loaded composite structures, as long as appropriate structural models are used to describe the material behavior. A CEL simulation was performed to replicate the experimental observations reported in a paper published Gargano et al. (2019) [7], which involves a CFRP laminate subjected to blast loading. Simpler uncoupled simulations were also performed according to the uncoupled scheme to compare the performance of coupled and uncoupled methods. This paper is organized as follows. Section 2 presents the CEL and uncoupled approaches considered to carry out the case study, along with the experimental campaign selected in the literature for validation of the numerical simulations, the structural models and the parameters involved. The results are presented in Section 3, and the main findings are discussed in Section 4. Finally, conclusions are drawn in Section 5.

## 2. Materials and Methods

The experimental campaign considered to validate the proposed approaches is presented in Section 2.1. The two numerical approaches employed to model the blast load event and the two material models used to predict the composite plate behavior are also introduced in Section 2.2 and Section 2.3. Both methodological and theoretical information, as well as specific details related to the LS-DYNA software package, which was employed to carry out the simulations, are described in this section.

### 2.1. Validation Case Study

The case study considered in this work to validate the numerical simulations was taken from the work of Gargano et al. (2019) [7]. This scenario involves the detonation of a spherical type-4 plastic explosive charge of 100 g at a stand-off distance of 0.4 m from an initially flat, quadrangular composite plate. The carbon material utilized in the laminates consisted of a single-ply plain woven fabric with an areal density of 600 $g/m^{2}$. To create the laminates, seven plies of this carbon fabric were stacked, aligning the warp tows in a consistent direction to achieve a balanced cross-ply fiber pattern [0/90]. The fabric preforms underwent an infusion process with liquid polyester at room temperature, employing the vacuum bag resin infusion technique (VRI). For the polyester resin (specifically, polyplex isophthalic resin 45 provided by Nuplex Composites), a catalyst was added, consisting of 1 wt% methyl ethyl ketone peroxide (MEKP) solution (40 wt% MEKP in dimethyl phthalate) known as SPV 1265, also from Nuplex Composites. Subsequent to the VRI process, the laminates underwent a gelation and partial curing stage at 20 °C over one day, followed by a post-curing process at 80 °C for one hour. The laminate exhibited a consistent thickness of 4.2 ± 0.1 mm and possessed a fiber volume content measuring 50 ± 2%. The composite plate was made up of seven 0.6 mm thick plies, and the in-plane dimensions were 275 × 275 mm^2^. A steel window frame was used to fix the plate, leaving an exposed area of 250 × 250 mm^2^. The frame was lined with EPDM 414 foam, which separated the steel frame and the composite plate and created a simply supported boundary condition for the blast-loaded structure. The interested reader is referred to the work reported in Ref. [7] for additional details about the experimental setup.

### 2.2. Uncoupled Approach

The uncoupled approach consists of simulating the blast response of structures using a two-step procedure. First, the pressure load acting on the rigid structure is predicted using analytical or empirical models or by conducting Eulerian simulations. Then, the pressure load is applied to the deformable structure in a standard FE simulation. The uncoupled approach selected in this work is commonly known as *the pure Lagrangian approach* and is based on the usage of analytical and empirical models to compute the pressure load from the explosion. Specifically, the blast pressure is computed using the ConWep method, which is based on the Kingery–Bulmash (KB) equations [15]. These equations were obtained by fitting experimental results and only require the scaled distance as an input value, which is defined according to Equation (1) [16,17]:

(1) $$Z=\frac{R}{\sqrt[{3}]{W_{TNT}}}$$

where *R* is the stand-off distance, i.e., the distance from the center of the high explosive (HE) spherical charge to the point of interest, while $W_{TNT}$ is the TNT-equivalent weight of the considered explosive material. The KB relationships employed in this work are valid within the range of $0.147\;m\cdot {\mathrm{kg}}^{-1/3}<Z<40\;m\cdot {\mathrm{kg}}^{-1/3}$. Thus, the predicted blast parameters may not be accurate enough in close-range scenarios characterized by scaled distances below the 0.147 $m\cdot {\mathrm{kg}}^{-1/3}$ limit [18]. The analytical pressure applied to the target structure is computed according to Equation (2) [19]:

(2) $$P=P_{R}\cdot cos^{2}\theta +P_{I}\cdot \left(1+cos^{2}\theta -2cos\theta \right)$$

where $P_{R}$ and $P_{I}$ are the incident and reflected pressure values that are estimated by the KB equations, respectively; while θ is the angle of incidence. The analytical pressure is computed at each step of the solution for each loaded element, since the θ variable varies during the analysis as the structure deforms and the values of $P_{R}$ and $P_{I}$ depend on the position of the target elements with respect to the detonation point and the time passed from the detonation. The reader is referred to [14] for a more detailed discussion of this methodology. The blast load predicted using the pure Lagrangian method is commonly considered accurate and efficient within the validity limits of the model, but no FSI effects can be accounted for using this method.

The model described above is implemented in LS-DYNA through the keyword *LOAD_BLAST_ENHANCED. The value BLAST = 2 was used to specify that the charge considered in the simulations was spherical. According to the equations proposed in Ref. [20], 168 g of TNT equivalent was considered to guarantee equivalence in terms of blast pressure prediction. Hence, given that the stand-off distance was 0.4 m, the scaled distance value associated with the explosion was $Z=0.72\;m\cdot {\mathrm{kg}}^{-1/3}$. This value lies within the validity range of the selected uncoupled method.

**Figure 1:**
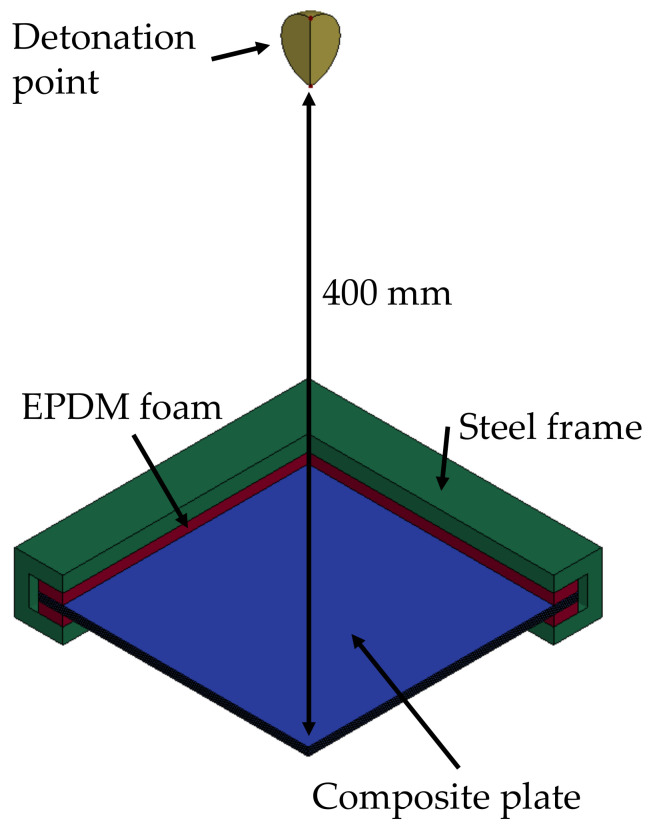
Schematic representation of the model developed within the uncoupled scheme.

Figure 1 shows a schematic representation of the model developed within the uncoupled scheme described above. Double symmetry was applied to reduce the computational time.

### 2.3. Coupled Eulerian–Lagrangian Approach

The CEL method consists of a single simulation wherein the structure and the fluids are simultaneously present. The structural response is described using the FE method, while the fluids are usually modeled using the finite difference scheme. Coupling algorithms are used to connect the structural and fluid domains. This also allows the method to account for FSI effects.

The thermodynamic state evolution of the HE material after detonation is typically evaluated through the Jones–Wilkins–Lee (JWL) equation of state shown in Equation (3) [6]:

(3) $$P\left(V,e\right)=A\left(1-\frac{\omega V_{0}}{VR_{1}}\right)\exp\left(\frac{-VR_{1}}{V_{0}}\right)+B\left(1-\frac{\omega V_{0}}{VR_{2}}\right)\exp\left(\frac{-VR_{2}}{V_{0}}\right)+\omega \rho e$$

where *V* is the inverse of density (ρ), *e* is the internal energy, *A* and *B* are parameters with units of pressure, ω is the Grüneisen coefficient, and $R_{1}$ and $R_{2}$ are dimensionless parameters. The pressure wave produced by the detonation of the HE charge propagates in the surrounding material and reaches the target structure, exerting the pressure load. Hence, the surrounding material needs to be modeled to describe the shockwave propagation up to the target structure. Typically, the behavior of the air domain is governed by the ideal gas equation of state [21].

To reduce the computational effort of this method, in this work, a *hybrid CEL approach* was employed; that is, blast-wave generation and propagation far from the plate were described using KB empirical equations, while close to the structural domain, the air was explicitly modeled to keep track of possible FSI effects. A layer of receptor elements was used to link the empirical description of the blast wave to the fluid domain or, in other words, to let the pressure wave enter and propagate inside the explicitly modeled fluid domain. The distance between the receptor elements and the detonation point was 380 mm. Thus, the KB equations were used to compute the pressure load at a scaled distance of $Z=0.69\;m\cdot {\mathrm{kg}}^{-1/3}$. This value lies within the range of validity of the KB equations. Hence, this modeling choice did not limit the validity of the coupled approach but allowed for a reduction in the computational time required to propagate the blast wave far from the structure.

Specifically, in this work, the air domain was modeled with solid hexahedral elements with a characteristic dimension of convergence of 1 mm. The formulation selected in this work is the solid-section ELFORM = 5, which identifies 1-point arbitrary Lagrangian–Eulerian (ALE) elements. The keyword *ALE_REFERENCE_SYSTEM_GROUP was employed to model the behavior of the ALE elements. The PRTYPE = 8 mesh smoothing option was considered, along with an initial mesh remapping factor of EFAC = 1, which was used to force the pure Eulerian behavior of the elements describing the fluid domain. The card *CONTROL_ALE was included with the following parameters: METH = 3 as an advection method, AFAC = −1 to turn off the smoothing weight factor and EBC = 0 to set the flow-out boundary conditions. Finally, the reference pressure value applied to the free surfaces of the ALE mesh boundary (*PREF*) was set to 101.325 Pa. The ideal gas equation of state (*MAT_009) with $1.225\;\mathrm{kg}/m^{3}$ density and $1.8\cdot 10^{-5}\;\mathrm{Pa}\cdot s$ dynamic viscosity was used to model the behavior of air.

A segment set was created to specify the elements acting as receptors for the blast wave. The pressure load applied to the receptors was computed through the keyword *LOAD_BLAST_ENHANCED, which was described by the same parameters already mentioned in Section 2.2. The card *LOAD_BLAST_SEGMENT_SET was used to apply the pressure load to the receptor elements. The interaction between the structural and fluid domains was driven by the *CONSTRAINED_LAGRANGE_IN_SOLID card. The CTYPE = 4 fluid–structure coupling method, which is a penalty coupling for solid elements without erosion, was adopted.

**Figure 2:**
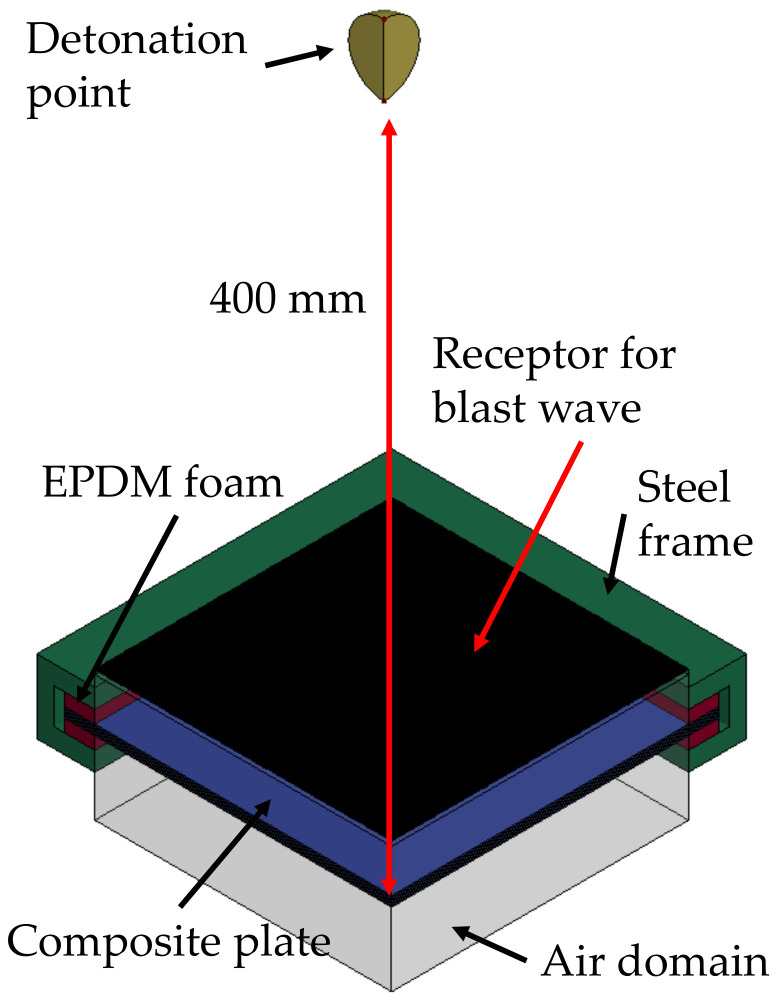
Schematic representation of the model developed within the hybrid CEL approach. The transparent block indicates the fluid domain.

The model developed within the hybrid CEL approach is presented in Figure 2. Double symmetry was applied to further reduce the computational time.

### 2.4. Structural Components

The target structure was modeled using the formulation of Lagrangian elements. The composite material was modeled with a macrohomogeneous discretization, i.e., each ply was modeled with a layer of elements. The intralaminar and interlaminar properties of the composite material were both considered in the analyses. The hourglass deformation modes in the structural parts were controlled by applying the Flanagan–Belytschko stiffness form with 0.03 hourglass coefficient according to the work of Maio et al. (2013) [22].

The composite plate was modeled using two different approaches. In one case, the MAT_054 material model was used with reduced-integration thick shell elements; in the other, the MAT_162 material model with reduced-integration solid elements was used [21,23].

In the model using the MAT_054 material model, each ply was modeled with a layer of thick shell elements with a 2 mm reference dimension, as suggested in the work reported in [7], and one element was modeled according to the thickness. The intralaminar mechanical properties were based on the Hashin failure criteria [24], and the woven composite behavior was introduced in the analysis with the 2WAY=1 flag in the material keyword. The equations governing ply failure are reported in Table 1. The main non-default parameter used in this work for this material model is reported in Table 2 according to those reported in [7]. In Table 2, *DFAILT* and *DFAILC* are intentionally set large values to avoid elemental erosion, similarly to the conditions assumed in the simulations reported in [7]. In addition, *SC* was set to a high value, and the shear coupling parameter (β) in the tensile failure modes was set to zero in order to neglect the shear contribution to failure according to Ref. [7].

**Table 1:** Woven composite failure criteria for MAT_054.

Failure Mode	Criteria
Tensile failure	(4) ${\left(\frac{\sigma_{a}}{X_{T}}\right)}^{2}+\beta {\left(\frac{\sigma_{ab}}{S_{C}}\right)}^{2}\geq 1$
	(5) $$\;{\left(\frac{\sigma_{b}}{Y_{T}}\right)}^{2}+\beta {\left(\frac{\sigma_{ab}}{S_{C}}\right)}^{2}\geq 1$$
Compressive failure	(6) ${\left(\frac{\sigma_{a}}{X_{C}}\right)}^{2}\geq 1$
	(7) $${\left(\frac{\sigma_{b}}{Y_{C}}\right)}^{2}\geq 1$$
Shear failure	(8) ${\left(\frac{\sigma_{ab}}{S_{C}}\right)}^{2}\geq 1$

Since MAT_054 only accounts for intralaminar damage of the composite, the interlaminar behavior was modeled with a contact interaction between adjacent plies. This interaction is based on the cohesive zone model (CZM) theory [25,26] and is applied using the keyword *CONTACT_AUTOMATIC_SURFACE_TO_SURFACE_TIEBREAK. This contact algorithm keeps the corresponding nodes between adjacent layers connected until failure occurs; after that, the interaction is turned into a simple surface-to-surface contact between the plies. Equation (9) defines the quadratic criterion governing the failure, which accounts for both the normal ($\sigma_{n}$) and shear ($\tau_{s}$) interlaminar stresses. The maximum allowable stresses are reported in Table 3 according to [7].

(9) $${\left(\frac{\sigma_{n}}{NFLS}\right)}^{2}+{\left(\frac{\tau_{s}}{SFLS}\right)}^{2}\geq 1$$

The other strategy employed to model the composite panel exploited a built-in material model identified as MAT_162 [21]. This model has been employed in the literature to study impact damage and dynamic loadings on composite structures [27,28,29]. Among its advantages, the interlaminar damage, i.e., delamination, is automatically considered without requiring additional features for the interply properties. However, the drawbacks of this material model are the high number of required parameters to account for the damage criteria and the delamination criterion, which requires each layer to be modeled with three elements according to the thickness. This results in a large number of elements, increasing the computational cost of the simulation if each single layer is modeled. As three elements are required based thickness, the mesh size in the models with this material model was reduced to 1.4 mm to reduce the aspect ratio of the elements. The input parameters for these analyses are reported in Table 4.

The damage initiation criteria are shown by the equations in Table 5, which depend on the parameters reported in Table 4. Moreover, $S_{aFS}=S_{FS}$, $S_{bFS}=S_{FS}\cdot S_{bT}/S_{aT}$ and $S_{SRC}$ is defined as $S_{SRC}=E_{C}\cdot tan\phi \langle -\epsilon_{c}\rangle$, where ϕ is the Coulomb friction parameter, which is used to include the effect of compressive stress on shear strength. The damage thresholds ($r_{j}$ where 7≤j≤13) have initial values equal to 1 before damage is initiated, and they are updated due to damage accumulation in the associated damage modes. It is worth noting that the presence of Macaulay brackets (〈〉) means that if the strain value is negative, then it would be considered equal to zero. The fiber modes, i.e., tension and compression along the warp and weft direction of the woven material, are based on Hashin’s criteria [24] and are generalized to account for fiber damage in terms of strain components for a plain weave layer; in addition, in the compressive criteria, the compressive strain based on thickness is also accounted for. The crush mode considers the stress acting in the thickness direction. The two matrix failure modes account for in-plane and out-out-plane shear damage, the latter of which is related to delamination in this material model.

**Table 5:** Woven composite failure criteria for MAT_162.

Failure Mode	Criteria
Tension–shear fiber mode	(10) $f_{7}-r_{7}^{2}={\left(\frac{E_{a}\langle \epsilon_{a}\rangle }{S_{aT}}\right)}^{2}+{\left(\frac{G_{ca}\epsilon_{ca}}{S_{aFS}}\right)}^{2}-r_{7}^{2}=0$
	(11) $$f_{8}-r_{8}^{2}={\left(\frac{E_{b}\langle \epsilon_{b}\rangle }{S_{bT}}\right)}^{2}+{\left(\frac{G_{bc}\epsilon_{bc}}{S_{bFS}}\right)}^{2}-r_{8}^{2}=0$$
Compression fiber mode	(12) $f_{9}-r_{9}^{2}={\left[\frac{E_{a}\left(-\epsilon_{a}-\langle \epsilon_{c}\rangle \frac{E_{c}}{E_{a}}\right)}{S_{aC}}\right]}^{2}-r_{9}^{2}=0$
	(13) $$f_{10}-r_{10}^{2}={\left[\frac{E_{b}\left(-\epsilon_{b}-\langle \epsilon_{c}\rangle \frac{E_{c}}{E_{b}}\right)}{S_{bC}}\right]}^{2}-r_{10}^{2}=0$$
Crush mode	(14) $f_{11}-r_{11}^{2}=\left(\frac{E_{c}\langle -\epsilon_{c}\rangle }{S_{FC}}\right)-r_{11}^{2}=0$
In-plane matrix failure mode	(15) $f_{12}-r_{12}^{2}=\left(\frac{G_{ab}\epsilon_{ab}}{S_{ab}}\right)-r_{12}^{2}=0$
Parallel matrix failure mode	(16) $$\begin{array}{c} f_{13}-r_{13}^{2}=S^{2}\left[{\left(\frac{E_{c}\langle \epsilon_{c}\rangle }{S_{cT}}\right)}^{2}+{\left(\frac{G_{bc}\epsilon bc}{S_{bc0}+S_{SRC}}\right)}^{2}+{\left(\frac{G_{ca}\epsilon ca}{S_{ca0}+S_{SRC}}\right)}^{2}\right]-r_{13}^{2}=0 \end{array}$$

Four damage variables ($\omega_{i}$) degrade the stiffness properties of a composite depending on the encountered failure criteria. The progressive damage model proposed by Matzenmiller [33] correlates the compliance matrix with the damage variable, which, for an individual failure mode *j*, is shown in Equation (17):

(17) $$\omega_{i}=1-\exp\left(\frac{1-r_{j}^{m_{j}}}{m_{j}}\right)$$

where $r_{j}$ is the damage threshold as a function of the strain, and $m_{j}$ is one of four softening parameters controlling the compressive fiber failure mode in direction *a* (1), the tensile and compressive fiber failure mode in direction *b* (2), the softening associated with the fiber crush mode (3) and the in-plane and out-of-plane matrix failure modes (4). The values considered in this work for the softening parameters (*AM*) are reported in Table 4. For an in-depth discussion of this material model, the reader is referred to the work of Gama (2014) [31].

In order to reliably represent the plate response to the blast loading condition, the whole experimental setup needs to be modeled, as the results are significantly influenced by the boundary condition [34]. On the one hand, the steel frame was modeled as purely elastic, with the following properties: $7800\;\mathrm{kg}/m^{3}$ density, 203GPa Young’s modulus and 0.3 Poisson’s ratio. Regarding the EPDM 414, i.e., the soft foam lining the steel frame, to the best of our knowledge no, data are available in the literature about this foam, and none were specified in [7]; for this reason, a different foam taken from the work of Zhang et al. (2014) [35] is considered in our case. On the other hand, fully integrated solid hexahedral elements with a characteristic dimension of 2.5 mm are employed to model the foam material. In order to avoid excessive deformation and unstable numerical analysis, erosion was added to the foam with the *MAT_ADD_EROSION keyword, and the erosion criterion is governed by element deformation and occurs after a maximum effective strain equal to 10. The material constitutive behavior is implemented with the LS-DYNA keyword MAT_057, which is a law dedicated to highly compressible low-density foams, and the input parameters are reported in Table 6. The interested reader is referred to the LS-DYNA keyword user’s manual (Vol. II) for a more detailed description of the model [21].

## 3. Results

**Figure 3:**
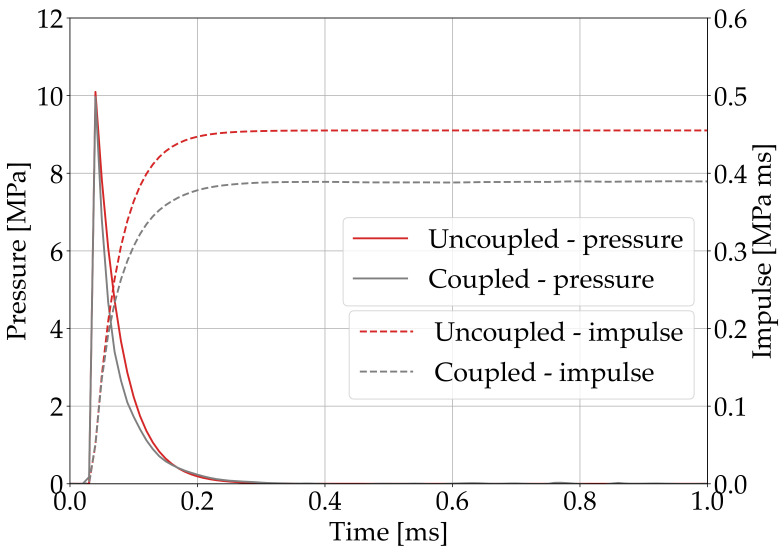
Pressure and impulse time histories predicted in the uncoupled and coupled simulations.

Figure 3 shows the pressure and impulse time histories obtained from the uncoupled and and CEL simulations. The curves are identical for both investigated material models. For this reason, only the results of the MAT_162 analyses are reported. The peak pressure in the CEL simulations was lower than in the uncoupled analyses. This is determined by FSI effects and adheres to the physics of the problem. FSI is also responsible for the faster pressure decay in the CEL simulations. As a result, the impulse curves slightly differed in the simulations; that is, FSI reduces the impulse imparted to the plate. The peak pressure values were 10.1 MPa and 9.8 MPa in the uncoupled and coupled simulations, respectively, while the maximum impulses were 458Pa·s and 408Pa·s, respectively. Since no experimental measurements were reported in Ref. [7], no further considerations can be provided to support the accuracy of the load predictions.

**Figure 4:**
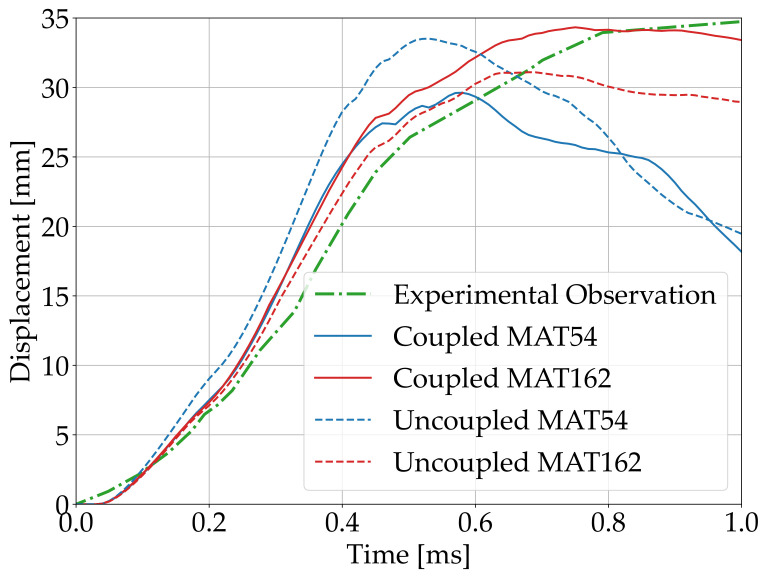
Displacement time histories of the plate center.

Figure 4 shows the displacement time histories of the central point of the plates. The maximum displacement predicted in the CEL simulations was 29.6 mm and 34.4 mm for MAT_54 and MAT_162, respectively, while these values were 33.5 mm and 31.1 mm, respectively, in the uncoupled simulations. The maximum experimentally observed displacement was 34.7 mm [7]. It turned out that the displacement time histories predicted in the coupled simulations better adhered to the experimental observation. The plot represented in the Figure was limited to 1 ms because within that time frame, the composite plate reached the maximum displacement both in the experimental and numerical tests. Moreover, the damage in the numerical simulations did not evolve after that time range. Note that in all the simulations and in the experimental test, the composite plate underwent elastic recovery after 1 ms [7].

**Figure 5:**
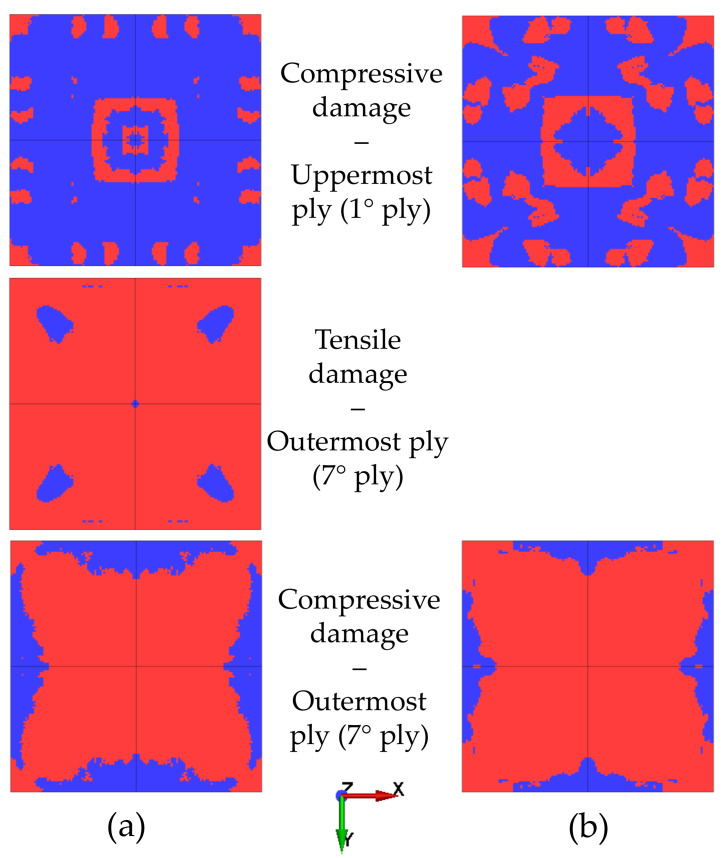
Intralaminar damage from the uncoupled (**a**) and coupled (**b**) simulations conducted using MAT_54.

**Figure 6:**
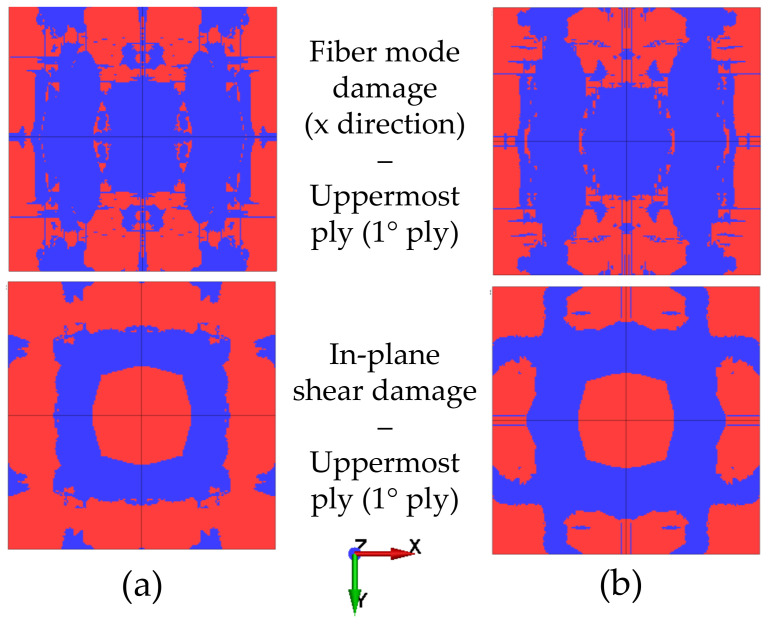
Intralaminar damage from the uncoupled (**a**) and coupled (**b**) simulations conducted using MAT_162.

Figure 5 and Figure 6 show the intralaminar damage observed in the analyses with MAT_54 and MAT_162, respectively. In both figures, subfigure (a) is related to the uncoupled simulations, while subfigure (b) shows the results of the coupled approach. Red areas represent the non-damaged material, while blue parts represent the elements that failed in a specific lamina, i.e., those in which a damage criterion was met. In the figures, *uppermost ply* is used to identify the ply facing the explosion, while the *outermost ply* is the one on the other side of the composite plate. Note that the two material models express the damage variable in a different manner; that is, MAT_54 allows tensile and compressive damage to be distinguished, whereas the two principal directions cannot be distinguished, as fibers are found along both directions in woven composites. Instead, MAT_162 accounts for different directions, but it does not discriminate between tensile and compressive fiber modes. These considerations show that the elements in the uppermost ply in Figure 6 most likely failed due to compressive damage, since that behavior was observed in the analyses conducted with MAT_54 (Figure 5). Moreover, although MAT_162 accounts for different directions, only the fiber-mode damage along the x direction is shown because the symmetry of the problem implies that the same pattern also characterizes the y direction. Shear damage was purposely neglected in the analyses conducted with MAT_54 according to the modeling method suggested in Ref. [7]. The results were comparable within the analyses carried out considering the same material model, meaning that the two methodologies are reliable for investigation of damage and failure of blast-loaded composite structures. The minor differences that can be seen when comparing the figures occurred due to the slightly different displacement predicted in the simulations; that is, the plates that underwent a higher degree of deflection showed a larger damaged area. The results are further discussed in Section 4.

**Figure 7:**
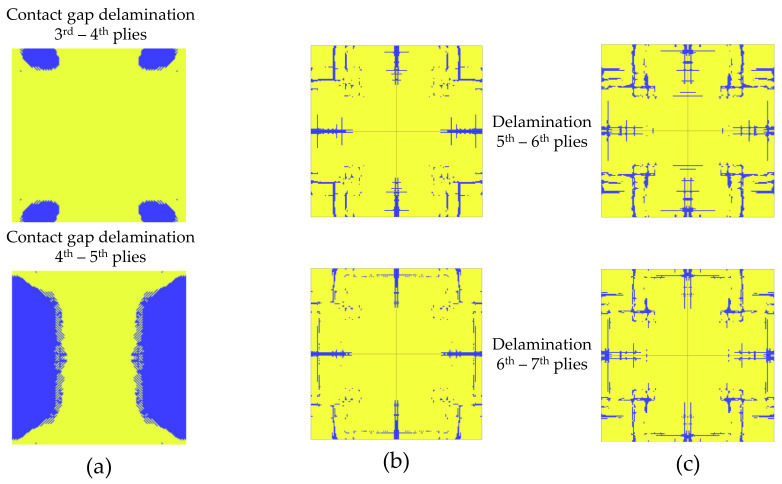
Interlaminar damage. (**a**) Uncoupled simulation conducted with MAT_54; (**b**) uncoupled simulation conducted with MAT_162; (**c**) coupled simulation conducted with MAT_162.

The interlaminar damage is shown in Figure 7. The area where the delamination occurred between two adjacent plies is marked in blue. Only the interlaminar damage observed in the uncoupled analysis conducted with MAT_54 and in the simulations conducted with MAT_162 are reported, as no delamination was detected in the coupled simulation conducted with MAT_54.

## 4. Discussion

The pressure load predicted using the uncoupled and coupled methods (Figure 3) was similar, except for the slightly lower pressure peak and impulse in the CEL simulations. This was expected, since CEL methods account for FSI effects [11]. The uncoupled method is less computationally demanding than the coupled method, even though the former cannot describe thermal and blast secondary effects, which can only be considered in coupled simulations at the expense of computational efficiency. It is worth underlining that the peak pressure and impulse exerted on the plate were not experimentally measured in Ref. [7]; hence, no further considerations can be provided to support the accuracy of the load predictions.

The displacement time histories of the plate center (Figure 4) were generally similar across all simulations. However, after reaching the maximum displacement, the plates described by MAT_54 showed a more pronounced elastic recovery. This can be explained by the different formulation of the material models. In fact, only the model using MAT_162 accounts for progressive damage. Hence, after reaching the maximum displacement, the elastic properties of the plates were severely degraded only in the simulations conducted with MAT_162. The qualitative comparison of the results obtained with the experimental curve reported in a paper by Gargano et al. (2019) [7] and in Figure 4 showed that neglecting the progressive failure led to a rough approximation and excessive elastic recovery. The behavior of the curves from obtained using MAT_162 seemed to match the experimental curves more closely, but at the same time, calibrating progressive failure parameters is an expensive process which is beyond the scope of this work. In addition, progressive failure might even be more crucial in real applications than in the laboratory test considered here. It is also worth noting that adopting MAT_54 resulted in smaller displacement under the CEL approach than under the uncoupled approach, while the opposite was observed in the simulations conducted with MAT_162. This difference may be related to the combination of several factors: (i) the interaction of the surrounding Eulerian mesh with the different material models, (ii) the different meshes and (iii) the FSI effects. However, this aspect is worth further investigations that are beyond the scope of this work.

The results of the intralaminar damage of the two material models were comparable with respect to compressive damage; that is, in all analyses, the uppermost ply experienced severe compressive damage. This adheres to the physics of the problem, since during deformation, a compressive state of stress is generated in the upper layers of the plate, resulting in compressive damage. Furthermore, the compressive strength of the carbon woven lamina was much lower than its tensile resistance. This provides further evidence that tensile damage was only detected in the outermost layers, especially in the last ply in the uncoupled analysis conducted with MAT_54. The same damage was not seen in the analysis conducted using the CEL method, probably because the plate did not deflect enough to generate a high level of tensile stress in the outermost layer. Even in the analyses conducted using MAT_162, no fiber-mode damage was seen in the outermost layers. In fact, in these simulations, damage mostly occurred in the uppermost layers that were facing the blast. It is interesting to note that in Figure 6, the fiber mode (compressive damage) was not the only damage that was predicted by the model; in-plane shear damage also appeared to contribute. This evidence implies that the approximation obtained by neglecting the shear contribution in the analyses conducted with MAT_54, as well as in Ref. [7], may be rough and lead to underestimation of the damage.

**Figure 8:**
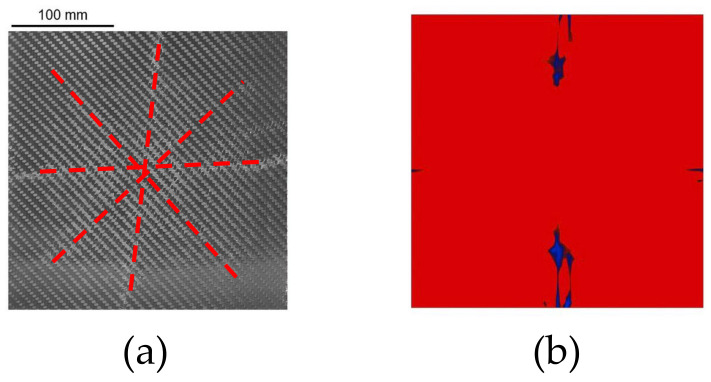
Intralaminar damage from the experimental test (**a**) and FE analysis (**b**) carried out in Ref. [7]. Reprint from Ref. [7].

The experimental and numerical intralaminar damage reported in the work of Gargano et al. is shown in Figure 8 [7]. The dashed red lines in Figure 8a highlight the area where damage was identified. Experimentally, damage was observed in the horizontal and vertical planes along the symmetry axes of the plate and along the two diagonals of the plate. The blue regions in Figure 8b were referred to as ply rupture in Ref. [7] and approximately reproduced the damage along one symmetry plane. Comparing these results to those presented in Figure 5 and Figure 6, the damage obtained in the analyses carried out in this work was extensive in the upper plies. However, in the early steps of the analyses, before the elements failed extensively, the damage nucleated along the oblique direction of the plate and propagated towards the symmetry planes of the plates later in the simulations. However, the oblique damage pattern was clearly visible in the outermost ply in the uncoupled analysis conducted with MAT_54, as shown in Figure 5a. Interestingly, the analyses conducted using MAT_54 predicted severe compressive damage in the clamped area, as well as in the outer plies, while considering MAT_162, similar damage was seen only in the upper plies. This damage is likely to be strongly related to the effect of the boundary conditions, which heavily influenced the type of damage, as well as the macroscopic behavior of the plate, e.g., the central-point displacement time history [34]. The damage in the clamped area was not observed in the experimental results, or it might not be as visible as the damage occurring closer to the center of the plate.

**Figure 9:**
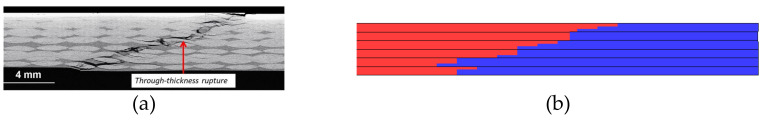
Cracking of the composite plate through the thickness. (**a**) Experimental observations from Gargano et al. (2019) (Reprint from Ref. [7]); (**b**) uncoupled and coupled numerical analyses conducted in this work using MAT_162.

Figure 9a shows an image reported in Ref. [7], where a crack propagated through the thickness during the blast event is noted. The same pattern was also seen in the analyses carried out in this work using MAT_162 (Figure 9b), where the blue areas failed by parallel matrix failure mode (Equation (16)), i.e., through thickness damage occurred.

**Figure 10:**
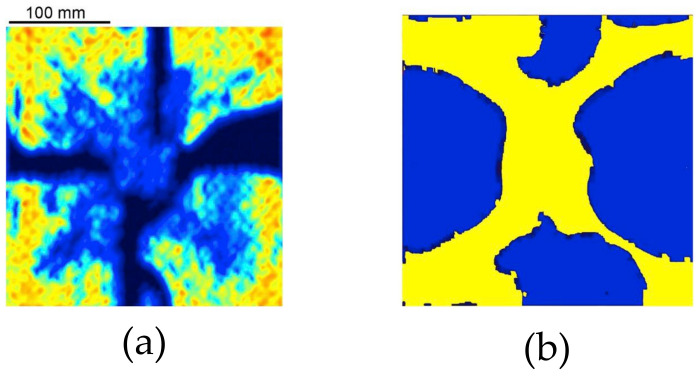
Interlaminar damage comparison from the experimental test (**a**) and FE analysis (**b**) reported by Gargano et al. (2019) (Reprint from Ref. [7]).

The experimental and numerical comparison of the interlaminar damage reported in Ref. [7] is shown in Figure 10. Figure 10a shows the experimental damage observed with an ultrasound technique, where the blue and black parts represent the interlaminar damage. The numerical interlaminar damage is also marked in blue color in Figure 10b. The experimental results retrieved from Ref. [7] are comparable to those observed in the analyses conducted employing MAT_162, but they differed from those obtained using MAT_54. In fact, in the case of the analyses conducted with MAT_162, delamination occurred especially along the symmetry axis, resembling the experimental observations shown in Figure 10a. Instead, the interlaminar damage obtained in the analysis conducted with MAT_54 were more similar to the numerical observations reported in Ref. [7] (Figure 10b). The difference is likely to be related to the different way the damage was accounted for in the two cases: in the models using MAT_162, delamination was considered with a damage criterion, while in the model with MAT_54, delamination was modeled with a contact interaction based on the cohesive zone model. The latter is the same method used in Ref. [7], explaining the similar results between the two simulations. It should also be noted that the boundary conditions and the experimental setup play a key role in the damage resulting from such experimental tests. Boundary conditions affect the overall behavior of the plate and the damage pattern [34]. It was noted that the delamination propagated significantly in the last stages of the simulations, when the plate had almost reached the maximum deflection and the foam was very compressed. Therefore, it can be concluded that reliably modeling boundary conditions is paramount to obtain accurate results in this type of analysis.

In summary, the two methods captured different damage patterns that matched the experimental observations.

## 5. Conclusions

In this work, we compared the performance of uncoupled and coupled methods for the study of blast-loaded composite plates. An experimental case study was selected from the literature for validation.

The following conclusions can be drawn:

- The exerted pressure and the resulting maximum deflection predicted in the simulations adhered to the experimental observations.
- The damage patterns identified in the simulations were comparable with the damage observed in the experiments.
- The analyses conducted with MAT_54 were found to be satisfactorily accurate, even though MAT_162 allowed for better matching with the experimental observations. However, MAT_54 is believed to provide the best tradeoff between ease of implementation and accuracy of the results. In fact, MAT_162 offers considerable freedom in calibrating the composite mechanical behavior, although a large number of parameters is required to calibrate damage initiation and propagation.
- Only the coupled method allows for comprehensive modeling of the blast event, also considering FSI, thermal effects and secondary blast effects, and can more accurately estimate the pressure load than uncoupled approaches. However, in most cases, the load predictions from uncoupled simulations are already sufficiently accurate, while the uncertainty associated with structural material parameters obscures the advantages that could be gained from utilizing more precise pressure loads.

Not all the open issues have been addressed yet. Improvements can be made in the representation of the blast-induced damage by improving and refining the modeling of the involved materials. For instance, the effect of the strain rate can be investigated, given the high strain rate involved under dynamic loading conditions, although this effect has resulted in conflicting results reported by different researchers. Furthermore, accurately representing the mechanical behavior of the other materials involved in the experimental test is regarded as crucial, as already underlined in the discussion.

## Figures and Tables

**Table 2:** Carbon–polyester laminate ply parameters for MAT_054 [7].

Material Property	LS-DYNA Symbol	Value
Density	RO	1600 $\mathrm{kg}/m^{3}$
In-plane longitudinal Young’s modulus	EA	55 GPa
In-plane transversal Young’s modulus	EB	55 GPa
Out-of-plane Young’s modulus	EC	7 GPa
In-plane Poisson’s ratio	PRBA	0.25
In-plane shear modulus	GAB	4.5 GPa
Out-of-plane shear modulus	GBC	1.8 GPa
Out-of-plane shear modulus	GCA	1.8 GPa
Woven composite failure criteria flag	2WAY	1
Maximum strain value for fiber tension	DFAILT	1
Maximum strain value for fiber compression	DFAILC	−1
Longitudinal compressive strength	XC	240 MPa
Longitudinal tensile strength	XT	680 MPa
Transversal compressive strength	YC	240 MPa
Transversal tensile strength	YT	680 MPa
Shear strength	SC	1000 MPa

**Table 3:** Properties of the contact interaction between adjacent plies [7].

Material Property	LS-DYNA Symbol	Value
Maximum normal stress	NFLS	60 MPa
Maximum shear stress	SFLS	60 MPa

**Table 4:** Carbon–polyester laminate ply parameters for MAT_162.

Material Property	LS-DYNA Symbol	Value	Ref.
Density	RO	1600 $\mathrm{kg}/m^{3}$	[7]
In-plane longitudinal Young’s modulus	EA	55 GPa	[7]
In-plane transversal Young’s modulus	EB	55 GPa	[7]
Out-of-plane Young modulus	EC	7 GPa	[7]
In-plane Poisson’s ratio	PRBA	0.25	[7]
Out-of-plane Poisson’s ratios	PRCA, PRCB	0.05	[7]
In-plane shear modulus	GAB	4.5 GPa	[7]
Out-of-plane shear moduli	GBC, GCA	1.8 GPa	[7]
Longitudinal tensile strength	SAT	680 MPa	[7]
Longitudinal compressive strength	SAC	240 MPa	[7]
Transversal tensile strength	SBT	680 MPa	[7]
Transversal compressive strength	SBC	240 MPa	[7]
Through the thickness tensile strength	SCT	50 MPa	[30]
Crush strength	SFC	700 MPa	[30]
Fiber-mode shear strength	SFS	120 MPa	[30]
Matrix-mode in-plane shear strength	SAB	80 MPa	[30]
Matrix-mode out-of-plane shear strength	SBC, SCA	60 MPa	[30]
Scale factor for residual compressive strength	SFFC	0.3	[30]
Coulomb’s friction angle	PHIC	10	[30]
Element-eroding axial strain	E_LIMT	3	[30]
Scale factor for the delamination criterion	S	1.1	[30]
Limit damage parameter for elastic modulus reduction	OMGMX	0.999	[30]
Element-eroding axial strain	E_LIMIT	3	[30]
Limit compressive relative volume for elemental erosion	ECRSH	0.001	[31]
Limit tensile relative volume for elemental erosion	EEXPN	3	[30]
Coefficient for the strain-softening property	AM1, AM2	1	[32]
Coefficient for the strain-softening property	AM3	0.35	[30]
Coefficient for the strain-softening property	AM4	0.3	[30]

**Table 6:** Parameters of the material of the foam [35] for MAT_057.

Material Property	LS-DYNA Symbol	Value
Density	RO	63 $\mathrm{kg}/m^{3}$
Young’s modulus	E	8.4 MPa
Hysteretic unloading factor	HU	0.25
Decay constant for creep unloading	BETA	5.0
Viscous coefficient for damping effects	DAMP	0.5
Shape factor for unloading	SHAPE	5.0
Stiffness coefficient for contact interface stiffness	KCON	1150 MPa

## Data Availability

Data available upon request.

## Abbreviations

The following abbreviations are used in this manuscript:

FSI	Fluid–structure interaction
HE	High explosive
JWL	Jones–Wilkins–Lee
KB	Kingery–Bulmash
CEL	Coupled Eulerian–Lagrangian
TNT	Trinitrotoluene
VRI	Vacuum bag resin infusion
